# Feasibility of neuromuscular training in patients with severe hip or knee OA: The individualized goal-based NEMEX-TJR training program

**DOI:** 10.1186/1471-2474-11-126

**Published:** 2010-06-17

**Authors:** Eva Ageberg, Anne Link, Ewa M Roos

**Affiliations:** 1 Department of Orthopedics, Clinical Sciences Lund, Lund University, Sweden; 2 Department of Health Sciences, Lund University, Sweden; 3 Institute of Sports Science and Clinical Biomechanics, University of Southern Denmark, Odense, Denmark

## Abstract

**Background:**

Although improvements are achieved by general exercise, training to improve sensorimotor control may be needed for people with osteoarthritis (OA). The aim was to apply the principles of neuromuscular training, which have been successfully used in younger and middle-aged patients with knee injuries, to older patients with severe hip or knee OA. We hypothesized that the training program was feasible, determined as: 1) at most acceptable self-reported pain following training; 2) decreased or unchanged pain during the training period; 3) few joint specific adverse events related to training, and 4) achieved progression of training level during the training period.

**Methods:**

Seventy-six patients, between 60 and 77 years, with severe hip (n = 38, 55% women) or knee OA (n = 38, 61% women) underwent an individualized, goal-based neuromuscular training program (NEMEX-TJR) in groups for a median of 11 weeks (quartiles 7 to 15) prior to total joint replacement (TJR). Pain was self-reported immediately after each training session on a 0 to 10 cm, no pain to pain as bad as it could be, scale, where 0-2 indicates safe, > 2 to 5 acceptable and > 5 high risk pain. Joint specific adverse events were: not attending or ceasing training because of increased pain/problems in the index joint related to training, and self-reported pain > 5 after training. The level of difficulty of training was registered.

**Results:**

Patients with severe OA of the hip or knee reported safe pain (median 2 cm) after training. Self-reported pain was lower at training sessions 10 and 20 (p = 0.04) and unchanged at training sessions 5 and 15 (p = 0.170, p = 0.161) compared with training session 1. There were no joint specific adverse events in terms of not attending or ceasing training. Few patients (n = 17, 22%) reported adverse events in terms of self-reported pain > 5 after one or more training sessions. Progression of training level was achieved over time (p < 0.001).

**Conclusions:**

The NEMEX-TJR training program is feasible in patients with severe hip or knee OA, in terms of safe self-reported pain following training, decreased or unchanged pain during the training period, few joint specific adverse events, and achieved progression of training level during the training period.

## Background

Exercise is recommended as first-line treatment of osteoarthritis (OA) of the hip and knee [1,2]. General exercise, such as aerobic training, and local exercise, such as strengthening training, show positive effects in terms of reduced pain and improved physical function [2-5]. Also at late stages of hip or knee OA, e.g., while on waiting list for total joint replacement (TJR), exercise is well tolerated [6].

Because muscle weakness of the lower extremity is common in people with OA, strength training has formed the cornerstone of specific training, with most research focusing on quadriceps strengthening in people with knee OA [7]. Improvements are achieved by strength training. However, because people with OA have functional instability [8] and defective neuromuscular function [7], it was recently suggested that neuromuscular exercises are important and may be needed to improve the effectiveness of training programs for these patients [7]. In patients with anterior cruciate ligament (ACL) injury, at high risk of knee OA [9], neuromuscular training has gained recognition over more traditionally used strength training in the past 10 to 15 years [10]. Neuromuscular training is also successfully used in the prevention of knee injuries [11].

There are some principal differences between neuromuscular and strength training programs. The aim of neuromuscular training programs is to improve sensorimotor control and achieve compensatory functional stability. Functional, weight-bearing exercises are used in various positions, resembling conditions of daily life and more strenuous activities. The quality of the performance in each exercise is emphasized and the level of training and progression is guided by the patient's neuromuscular function [12]. Strength training programs usually consist of non-weight-bearing exercises training isolated muscles selectively, and sometimes also weight-bearing exercises are used, involving multiple joints. The quantity of muscle output is emphasized and the level of training and progression is guided by the patient's one-repetition maximum [7].

A neuromuscular training method, based on biomechanical and neuromuscular principles [13], has been successfully used and evaluated in younger [12-15] and, recently, also in middle-aged [16] patients with knee injuries (ACL, meniscal injury), who are at high risk of OA [9,17]. These principles apply also to other knee injuries/diseases and to other joints in the lower extremities [12,14]. The aim of this study was to apply the principles of neuromuscular training to older patients with severe hip or knee OA. We hypothesized that the neuromuscular training method was feasible in these patients, determined as: 1) at most acceptable self-reported pain following training; 2) decreased or unchanged pain during the training period; 3) few joint specific adverse events related to training, and 4) achieved progression of training level during the training period.

## Methods

### Patients

Seventy-six patients (44 women) between 60 and 77 years old with severe primary OA of the hip (n = 38, 55% women) or knee (n = 38, 61% women), all assigned for TJR, were recruited from the Department of Orthopedics, Lund University Hospital during 2007-2009 (Table 1). Exclusion criteria were co-morbidities influencing physical activity (e.g., other musculoskeletal disorders, neurological diseases), TJR in any hip or knee in the last 12 months, dementia, and not understanding the Swedish language.

**Table 1:** Characteristics of the patients

Characteristic	Hip OA (n = 38)	Knee OA (n = 38)	All (n = 76)
Women (n (%))	21 (55)	23 (61)	44 (58)
Age (y), mean (SD)	67 (3.8)	69 (4.3)	68 (4.1)
BMI (kg/m^2^), mean (SD)	27.8 (4.3)	29.9 (4.5)	28.9 (4.5)

The research ethics committee at Lund University approved the study (LU 81/2006) and the participants signed a written informed consent.

### Neuromuscular training method

#### Principles of neuromuscular training

The neuromuscular training method, based on biomechanical and neuromuscular principles, aims to improve sensorimotor control and achieve compensatory functional stability. Sensorimotor control (also called neuromuscular control) is the ability to produce controlled movement through coordinated muscle activity, and functional stability (also called dynamic stability) is the ability of the joint to remain stable during physical activity [18]. The neuromuscular training method has been evaluated in younger patients with ACL injury [12-15] and in middle-aged patients with meniscectomy [16,19]. The biomechanical and neuromuscular principles have been described in detail previously [12-14,16]. These principles apply also to other knee injuries/diseases and to other joints in the lower extremities, since the training aims at resembling conditions in daily life and more strenuous activities [12,14].

The principles include the following: *Active movements in synergies *of all the joints in the injured extremity are included. The movements start with the uninjured extremity, initiating the normal movement and applying *bilateral transfer effect of motor learning *to the injured leg. To improve sensorimotor control, exercises are mainly performed in *closed kinetic chains *in different positions (e.g., lying, sitting, standing) in order to obtain low, evenly distributed articular surface pressure by *muscular coactivation*. The model emphasizes the enhancement of antigravity *postural functions *of weightbearing muscles and the provocation of *postural reactions *in the injured leg by using voluntary movements in the other lower extremity, trunk and arms. The goal is to obtain equilibrium of loaded segments in static and dynamic situations without undesirable compensatory movements, with the aim of acquiring postural control in situations resembling conditions of daily life and more strenuous activities. Thus, the *quality of the performance *in each exercise with an appropriate position of the joints in relation to each other (postural orientation), i.e., with the hip, knee and foot well aligned, is emphasized.

The level of training and progression is guided by the patient's neuromuscular function and with regard to the affected joint structures. Strength, coordination, balance, and proprioception are all included in the exercises. Although these aspects are all included, the main focus can be, e.g., balance in one exercise and strength in another. To achieve the desired requirement of postural activity, the patients perform the exercises in various positions, i.e., lying, sitting, and standing. Progression is provided by; varying the number of, direction, and velocity of the movements; increasing the load; and/or changing the support surface.

We have named the neuromuscular training method NEuroMuscular Exercise (NEMEX). Since the training method is based on principles, an ending is added after NEMEX to indicate the patient group to which the specific program applies. In this particular study, including patients assigned for TJR, the training program is called NEMEX-TJR.

#### The NEMEX-TJR training program

We have applied the principles of neuromuscular training in the NEMEX-TJR training program as follows: The training sessions consists of three parts: warming up, a circuit program, and cooling down. The warm-up period consists of ergometer cycling for 10 minutes. The circuit program comprises four exercise circles, including neuromuscular exercises with the key elements: core stability/postural function; postural orientation; lower extremity muscle strength; and functional exercises. The exercises are mainly performed in closed kinetic chains. Because muscle weakness of the lower extremity, particularly the quadriceps, is common in patients with OA, exercises in open kinetic chains are also used to improve muscle strength of the knee and hip muscles. One or two exercises are performed in each exercise circle. Each exercise is performed 2-3 sets * 10-15 repetitions, with rest corresponding to one set, between each set and exercise. The exercises are performed with both the non-affected and the affected leg, although focus is on the affected leg. To allow for progression, three levels of difficulty are given for each exercise. Progression is made when an exercise is performed with good sensorimotor control and good quality of the performance (based on visual inspection by the physical therapist) and with minimal exertion and control of the movement (perceived by the patient). The last part of the training program includes cooling down, and stretching exercises for the lower extremity muscles (10 minutes). The exercises in the three parts of the training program are given in the additional file 1.

Training took place in groups, under the supervision of an experienced physical therapist specializing in training of musculoskeletal disorders. On average, about 10 patients attended a training session. Patients continuously entered the group training, i.e., the group held both novice patients and those who had participated in several training sessions and, thus, were more familiar with the training. During each group training session, each participant was monitored individually so that the exercises were performed at a training level according to their neuromuscular function. The patients were offered 2 training sessions a week of 60 minutes each. The training sessions took place late morning/before noon, since patients with hip or knee OA often report more pain early morning and in the afternoon. The patients participated in the training until they underwent TJR. The number of weeks of training was dependent on the waiting list for surgery, and was not pre-defined in the study.

Pain is a major symptom for patients with hip or knee OA. Therefore, we included a scale for monitoring pain during training (additional file). The patients were told that pain was allowed up to 5 on a 0 to 10 scale during and after the training session [20]. They were also told that the day after training, pain should subside to "pain as usual". If pain did not subside, the level of training was reduced [20].

### Assessment

#### Self-reported pain

A visual analog scale (VAS) graded from 0 to 10 was used for patient-reported pain after each training session, where 0 is "no pain" and 10 "pain as bad as it could be" [20]. Pain up to 2 on the scale was considered "safe", pain up to a level of 5 was considered "acceptable", and pain above 5 was considered "high risk" [20]. The last consecutive 36 patients reported pain before and after each training session (both were rated after the training session, to avoid focusing too much on joint pain).

#### Joint specific adverse events

Joint specific adverse events were determined as: 1) not attending a training session and/or ceasing training because of increased pain/problems in the index joint related to the training; and 2) self-reported pain > 5 on the 0 to 10 scale after training. The number of weeks of training, and the number of training sessions were recorded. The reasons for not attending a training session, i.e., because of increased joint specific pain/problems related to the training or to other reasons (work-related, unrelated injury or illness, travel, personal reasons) were registered.

#### Progression of training level

The level of training (1, 2, or 3), based on the physical therapist's estimation of the average level of all exercises that a patient performed during a training session, was registered after each training session.

### Statistical analysis

There were no differences in results between men and women and they were, therefore, analyzed as one group. The median value of self-reported pain on the 0 to 10 scale was calculated for each patient. Self-reported pain at training sessions 1, 5, 10, 15 and 20 was used for comparisons over time. The Wilcoxon signed rank test was used for comparisons over time, and in this analysis the patients with hip and knee OA were analyzed together since there were no differences between the groups. There were too few patients with self-reported pain before training at training sessions 15 and 20 to allow for comparison with self-reported pain at the first training session. Therefore, in the analysis of self-reported pain before training over time, training session 1 was compared with training sessions 5 and 10, respectively, only. Spearman's rank correlation coefficient was used to study the relation between self-reported pain and number of weeks of training, number of training sessions, and level of training.

## Results

### Self-reported pain after training sessions

**Figure 1:**
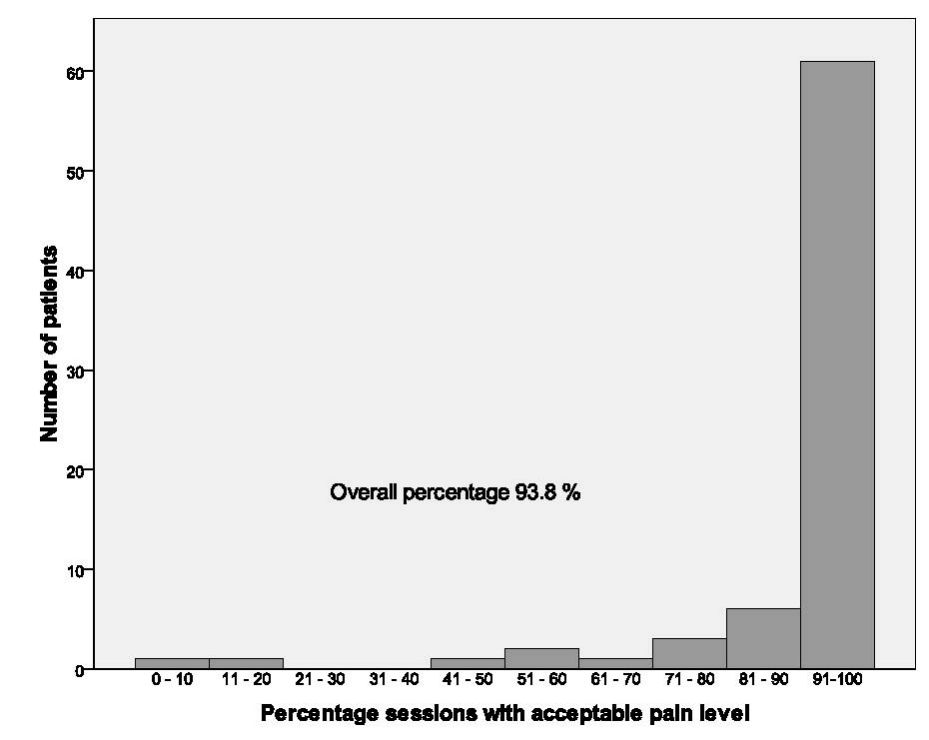
**Self-reported pain after training**. The percentage of training sessions with acceptable pain (≤ 5 on a 0 to 10 scale) in the patients, and overall percentage of training sessions with acceptable pain.

The median (quartiles) self-reported pain on the VAS after training was 2 cm (1 to 3) in patients with hip OA and 2 cm (1 to 4) in patients with knee OA. The overall percentage of training sessions with acceptable pain was 93.8 (Figure 1). Self-reported pain after training did not change from training session 1 (baseline) to training session 5 (median difference 0, p = 0.170, n = 65). Self-reported pain was lower at training session 10 than at training session 1 (median difference -0.5, p = 0.04, n = 53). There was no difference in self-reported pain at training session 15 compared with training session 1 (median difference 0, p = 0.161, n = 31). Self-reported pain was lower at training session 20 than at training session 1 (median difference -0.5, p = 0.04, n = 16).

### Self-reported pain before vs. after training sessions

In the subgroup of patients that reported pain both before and after training, there was no difference in median (quartiles) pain before vs. after training in those with hip OA (2.5 (2-3.25) vs. 2.75 (2-3), p = 0.357, n = 14) or in those with knee OA (3 (2-4) vs. 2.5 (1-4), p = 0.402, n = 22). There was no difference in self-reported pain before training at training session 1 compared with training session 5 (median difference 0, quartiles -1-1.75, p = 0.552, n = 32) or training session 10 (median difference 0, quartiles -2-1, p = 0.534, n = 26).

### Joint specific adverse events

#### Not attending or ceasing training

All patients were able to perform the training program at all sessions that they attended. There were no adverse events in terms of not attending a training session or ceasing training because of increased pain/problems in the index joint related to the training. All patients except 2 continued training until surgery. One of the 2 patients that discontinued training, ceased the training after 3 weeks (4 sessions) because of generally more pain (i.e., not joint specific) and disappointment since the surgery had been postponed for reasons not related to the joint disease or the training. The other patient did not turn up at the training after 2 weeks (3 sessions) for unknown reasons.

#### Self-reported pain > 5

One patient with hip OA and 2 patients with knee OA reported median pain above 5 on the 0 to 10 scale after training. Seventeen (22%) patients (hip OA n = 5, knee OA n = 12) reported pain > 5 after one or more training sessions. Six of these reported pain > 5 after one training session, and 11 patients reported pain > 5 after 2 or more training sessions.

### Progression of training level

**Figure 2:**
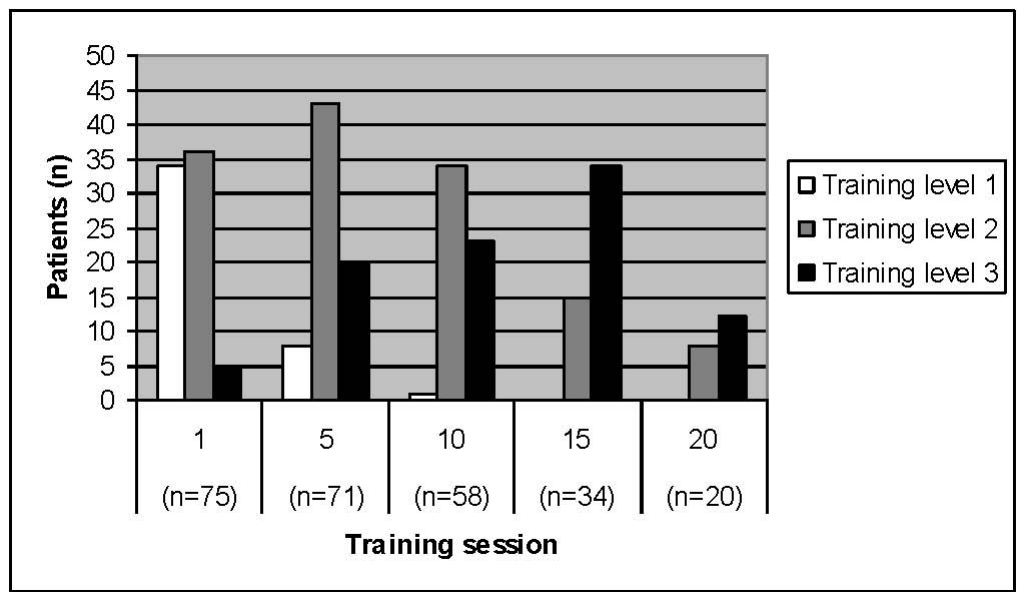
**Progression of level of training during the training period**. The number of patients training at levels 1, 2, and 3 at training sessions 1, 5, 10, 15 and 20. Note that the number of patients is reduced over time since the patients attended training until surgery, and the time from start of training to surgery was dependent on the waiting list and was not pre-defined in the study. Data for training level is missing for one patient.

The median (quartiles) training level was 2 (2 - 3) both in patients with hip OA and in those with knee OA. Progression of training was made from training session 1 to training session 5 (median difference 1, p < 0.001), and from training session 5 to training session 10 (median difference 0, p < 0.001). No progression was made from training session 10 to training session 15 (median difference 0, p = 0.083) or from training session 15 to training session 20 (median difference 0, p = 0.317). The number of patients training at the three different levels over time is given in Figure 2.

### Relation between self-reported pain and training

The median (quartiles) number of weeks of training was 11 (7 to 15) for patients with hip OA and 11 (8 to 14) for patients with knee OA. The median (quartiles) number of training sessions was 14 (10 to 18) for patients with hip OA and 13 (9 to 20) for patients with knee OA. In patients with hip or knee OA, respectively, there was no or low correlation between median self-reported pain after training and number of weeks of training (r_s _= -0.02, p = 0.90, and r_s _= -0.03, p = 0.86), number of training sessions (r_s _= -0.20, p = 0.23, and r_s _= -0.10, p = 0.57), and level of training (r_s _= -0.29, p = 0.08, and r_s _= -0.03, p = 0.87). This indicates that a higher number of training sessions (or weeks) and a higher level of training were not associated with more self-reported pain.

## Discussion

To our knowledge, this is the first study applying the principles of neuromuscular training, successfully used in younger and middle aged individuals with knee injury, to older people with severe hip or knee OA. We found that the individualized, goal-based neuromuscular training program, the NEMEX-TJR, was feasible in these patients in terms of safe self-reported pain after training, decreased or unchanged pain during the training period, few joint-specific adverse events, and achieved progression of training level during the training period.

General exercise, such as aerobic exercise and strength training, is generally recommended for improving overall health. General exercise is also recommended for people with OA, since such interventions show positive effects in terms of reduced pain and improved function [5,21-23]. The muscles contribute considerably to stabilizing the joints [24], and, therefore, training targeting the more specific needs related to the joint injury/disease has an important role in the treatment. It was suggested that joint-specific strengthening exercises are needed in the management of OA [4,22], and training programs have traditionally focused largely on strengthening lower extremity muscles [7]. However, neuromuscular training is successfully used in the prevention and treatment of knee injuries [10-12,16,25], i.e., in people at high risk of knee OA [9,17]. Such training may be important also for people with OA [7].

Neuromuscular training aims to improve sensorimotor control and obtain compensatory functional stability. Knee injury (ACL injury, meniscal injury, cartilage damage) leads to functional instability, i.e., a sudden loss of control of the injured joint in a weight-bearing position, and defective neuromuscular function (e.g., reduced strength and functional performance, differences in movement and muscle activation patterns, proprioceptive deficiency, and impaired postural control) [12,16,26]. These limitations are also reported by and observed in people with OA [7,8]. In our opinion, this warrants the use of neuromuscular training in people with OA.

Although most studies deal with general exercise in hip or knee OA, there are some studies on training in people with OA where functional exercises are used [27-29]. However, key components of neuromuscular training were lacking in these studies; principles of training, and level and progression of training, allowing an individualized approach, was described only in a case-report [27], and the quality of performance of exercises was not mentioned in any of these studies [27-29].

In accordance with that observed in people with knee injury, associated symptoms and functional limitations are heterogeneous in people with OA. Therefore, factors such as age, gender, previous and desired activity level, type of and severity of injury/disease, symptoms, and functional limitations are taken into account for each individual during neuromuscular training. An individualized approach to exercise based on various factors related to the joint and the individual is in line with that recommended for people with OA [4,22].

Both patients with knee OA and those with hip OA reported safe pain (median 2 cm) after training, a clear majority of the patients had acceptable pain after the training sessions, and there was no difference in self-reported pain after vs. before training sessions. We included a pain monitoring system, used previously in people with patellofemoral pain syndrome [20], to help the patients adjust training with regard to their perceived pain. The patients were told that pain was allowed up to 5 on a 0 ("no pain") to 10 ("pain as bad as it could be") scale during and after a training session, and the patients graded their pain on this 0 to 10 scale after each training session. They were also told that pain should subside to "pain as usual" the day after training, and if pain did not subside, progression of training was slowed down. The patients in our study did not report pain 24 hours after training, but we found that self-reported pain was decreased or unchanged over time, indicating that pain did subside. However, to ensure this, self-reported pain immediately after and 24 hours after training can be included in future studies. The effects of the NEMEX-TJR training program on pain and symptoms remain to be studied.

There were only few (n = 17, 22%) adverse events in terms of self-reported pain > 5 on the 0 to 10 scale. The highest number of patients (n = 6) with self-reported pain > 5 was observed after one training session only, and there were few patients (n = 4) with self-reported pain > 5 after more than 4 training sessions. Only two patients ceased training, one patient for unknown reasons and the other patient for reasons not related to the joint disease or training.

The NEMEX-TJR training program is divided into three levels of difficulty. All three training levels were used, and the training level was progressed over time. Because the training is individualized and goal-based, all patients did not progress to the third training level. The physical therapist supervising the training was experienced and specializing in training of musculoskeletal disorders, and we consider this a requirement in order to modify the exercises for each individual. Training took place in groups, to utilize the positive effects of group training in terms of more effective learning compared with individual practice sessions, and reduced costs [30]. As the exercises require little equipment, the training program can easily be employed in clinical settings and at home.

We found that the NEMEX-TJR training program was feasible in patients with severe hip or knee OA. Future studies should focus on the effects of the NEMEX-TJR on physical function and self-reported outcomes, dose-response, and comparison of this training program with other treatment. Another subject for further study may be training with the aim of postponing surgery. Such an approach would be of specific value for younger patients.

## Conclusions

The individualized goal-based neuromuscular training program, the NEMEX-TJR, is feasible in patients with severe hip or knee OA, in terms of safe self-reported pain following training, decreased or unchanged pain during the training period, few joint specific adverse events, and achieved progression of training level during the training period.

## Competing interests

The authors declare that they have no competing interests.

## Authors' contributions

EA contributed to the conception and design of the study, contributed to the design and adaptation of the neuromuscular training program, was responsible for acquisition, analysis and interpretation of data, drafted and critically revised the manuscript. AL contributed to the design and adaptation of the neuromuscular training program, collected the data, and critically revised the manuscript. ER contributed to the conception and design of the study, participated in interpretation of data, and critically revised the manuscript. All authors read and approved the final version.

## Pre-publication history

The pre-publication history for this paper can be accessed here:

http://www.biomedcentral.com/1471-2474/11/126/prepub

## Supplementary Material

Additional file 1 **NEMEX-TJR training program**. The NEuroMuscular EXercise training program (NEMEX) for patients with knee or hip OA assigned for total joint replacement (TJR), i.e., the NEMEX-TJR training program. Click here for file
