# Supplementary material for: Feasibility of neuromuscular training in patients with severe hip or knee OA: The individualized goal-based NEMEX-TJR training program
Source: BMC Musculoskelet Disord. 2010 Jun 17;11:126. doi: 10.1186/1471-2474-11-126 (PMC2896351; doi:10.1186/1471-2474-11-126)
Supplement: Additional file 1 — NEMEX-TJR training program. The NEuroMuscular EXercise training program (NEMEX) for patients with knee or hip OA assigned for total joint replacement (TJR), i.e., the NEMEX-TJR training program. [file 1471-2474-11-126-S1.PDF]

NEMEX-TJR training program

The NEuroMuscular Exercise training program for patients with knee or hip osteoarthritis (OA) assigned for total joint replacement (TJR), the NEMEX-TJR training program, is based on principles of neuromuscular training with the aim of improving sensorimotor control and achieving compensatory functional stability.

Training takes place in groups, under the supervision of an experienced physical therapist, and consists of three parts: warming up, a circuit program, and cooling down (described in detail below). A training session lasts 60 minutes. Patients continuously enter the group training, i.e., the group holds both novice patients and those who have participated in several training sessions and, thus, are more familiar with the training. The quality of the performance in each exercise with an appropriate position of the joints in relation to each other, i.e., with the hip, knee and foot well aligned, is emphasized (Figure 1).

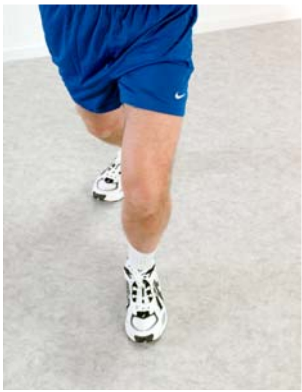

Figure 1A. “Knee-over-toe-position”, i.e., joints in lower extremity well aligned with appropriate position of knee over foot.

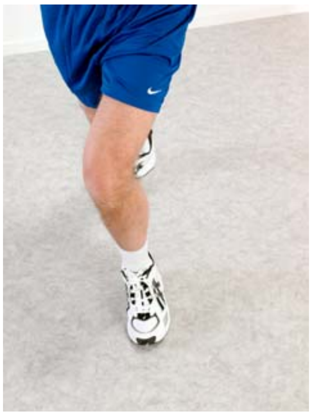

Figure 1B. “Knee-medial to-foot-position”, i.e., joints in lower extremity not aligned; knee is inappropriately placed medial to foot.

A visual analog scale (VAS) graded from 0 to 10 is used for monitoring pain during and after training, where 0 is “no pain” and 10 “pain as bad as it could be” (Figure 2) (Thomeé 1997). Self-reported pain up to 2 on the VAS is considered “safe”, pain up to a level of 5 is considered “acceptable”, and pain above 5 is considered “high risk”. The day after training, pain should subside to “pain as usual” and not increase over time. “Pain as usual” is defined as the pain level prior to exercise.

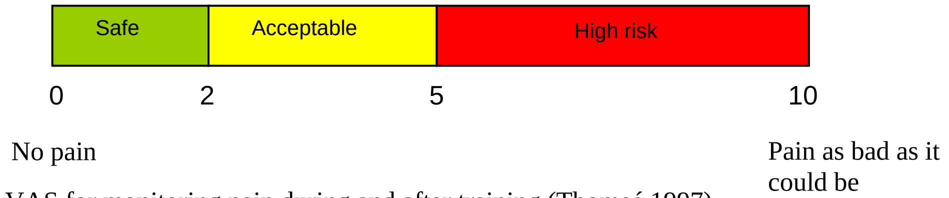

Figure 2. VAS for monitoring pain during and after training (Thomeé 1997).

Part 1. Warming up

The warm-up period consists of ergometer cycling for 10 minutes (Figure 3). The work load is set individually, and can be increased during the 10 minutes, aiming at reaching a perceived exertion of “somewhat hard”.

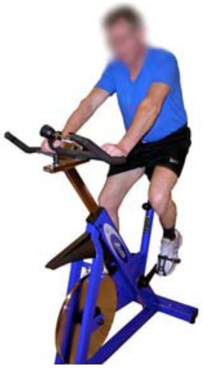

Figure 3. Warming up on a cycle ergometer.

Part 2. Circuit program

The circuit program comprises four exercise circles, with the key elements: core stability/postural function; postural orientation; lower extremity muscle strength; and functional exercises. Usually two exercises (A, B), sometimes one, are performed in each circle. Each exercise is performed 2-3 sets * 10-15 repetitions, with rest, corresponding to one set, between each set and exercise. Each patient is monitored individually, so that the exercises are performed at a training level according to their neuromuscular function. To allow for progression, three levels of difficulty are given for each exercise. Progression is provided by; varying the number of, direction, and velocity of the movements; increasing the load; and/or changing the support surface. Progression is made when an exercise is performed with good sensorimotor control and good quality of the performance (based on visual inspection by the physical therapist), and with minimal exertion and control of the movement (perceived by the patient). The exercises are performed with both the affected and the non-affected leg, although focus is on the affected leg. Some exercises are performed in front of a mirror, in order to provide visual feedback of the performance.

Exercise circle 1: Core stability/postural function

This circle includes exercises with focus on core stability and postural function.

Level 1
Exercises

A. **Pelvic-lift** with flexed knees and short lever arm, putting load on both legs (Figure 4)
B. **Sit-ups** with flexed knees, both legs on ball, arms along the sides (short lever arm) (Figure 5).

Level 2
Exercises

A. **Pelvic-lift** with semi-flexed knees and long lever arm, putting load on both legs.
B. **Sit-ups** with flexed knees, both legs on ball, arms crossed over chest (medium lever arm).

Level 3

Exercises

A. **Pelvic-lift** as above, alternately putting load on the affected and non-affected legs.
B. **Sit-ups** with flexed knees, both legs on ball, hands behind neck (long lever arm).

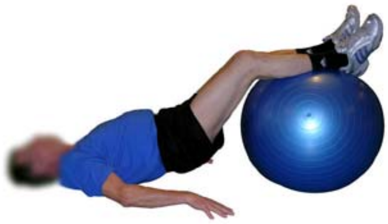

Figure 4. Pelvic-lift, level 1.

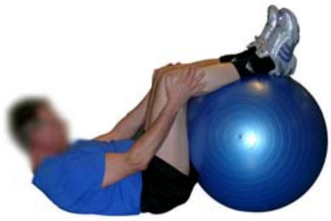

Figure 5. Sit-ups, level 1.

Exercise circle 2: Postural orientation

This circle includes exercises with emphasis on an appropriate position of the joints in relation to each other (postural orientation), i.e., with the hip, knee and foot joints well aligned (Figure 1).

Level 1

Exercises

A. **Slide-exercise forward-backward**: Standing, weight-bearing on one leg, other leg on sliding surface. Slide backwards – forwards with “sliding leg”, while flexing – extending the knee of the weight-bearing leg and keeping an appropriate position of the joints in relation to each other (Figure 6).
B. **Slide-exercise sideways**: Standing, weight-bearing on one leg, other leg on sliding surface. Slide sideways with “sliding leg”, while flexing – extending the knee of the weight-bearing leg and keeping an appropriate position of the joints in relation to each other.

Level 2

Exercises

A. **Slide-exercise forwards – backwards**: as above, standing on uneven surface (e.g., foam pillow or thick mattress).
B. **Slide-exercise sideways**: as above, standing on uneven surface (Figure 7).

Level 3

Exercises

A. **Forward lunge**: Standing position, take a large step forward and then return. Hand support for balance if needed (Figure 8).
B. **Sideway lunge**: Standing position, take a large step sideways and then return. Hand support for balance if needed.

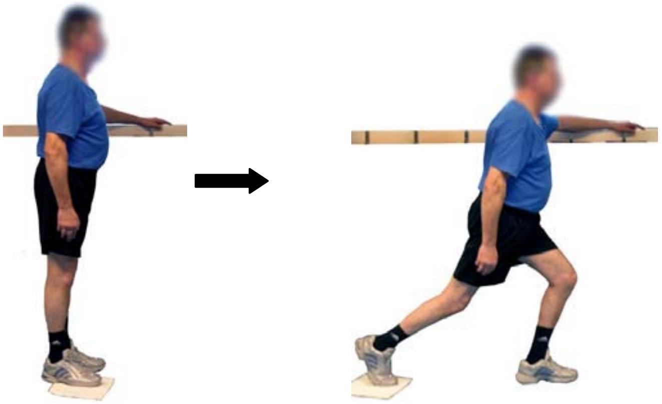

Figure 6. Slide-exercise backward-forward, level 1.

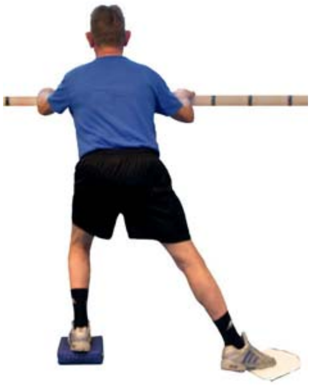

Figure 7. Slide-exercise sideways, level 2.

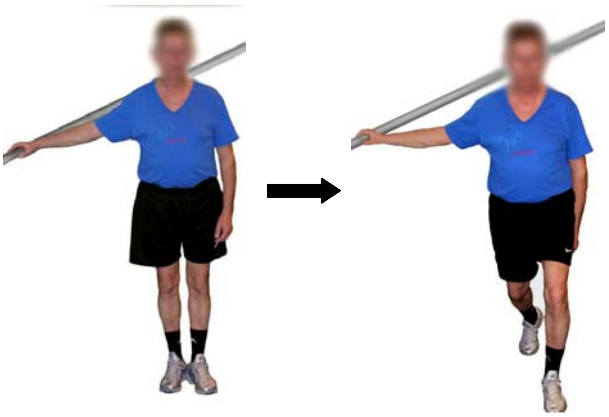

Figure 8. Forward lunge, level 3.

Exercise circle 3: Lower extremity muscle strength

This circle includes exercises in open and closed kinetic chains to improve strength of hip and knee muscles.

Level 1

Exercises

A. **Hip abductors/hip adductors**: Standing on one leg, rubber band other leg. Pull rubber band out (hip abductors) and in (hip adductors). Make sure there is tension in the rubber band also in resting position. Focus is on the hip abductors of the standing leg, keeping an appropriate position of the joints in the lower extremity in relation to

each other (Figure 1) and in relation to the trunk, i.e., without lateral displacement of the hip-pelvis region.

B. **Knee extensors/knee flexors**: Sitting position. Rubber band around one foot. Pull rubber band forward (knee extensors) and backwards (knee flexors). Make sure there is tension in the rubber band also in resting position.

Level 2

Exercises

A. Hip abductors/hip adductors: As above, rubber band with increasing resistance (Figure 9).

B. Knee extensors/knee flexors: As above, rubber band with increasing resistance (Figures 10-11).

Level 3

Exercises

A. **Hip abductors/hip adductors**: As above, standing on uneven surface (e.g., foam pillow or thick mattress).

B. **Knee extensors/knee flexors**: As above, rubber band with increasing resistance.

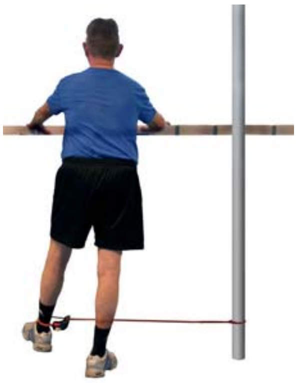

Figure 9. Hip abductors, level 2.

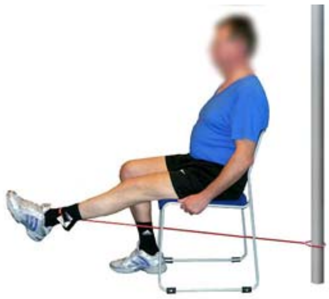

Figure 10. Knee extensors, level 2.

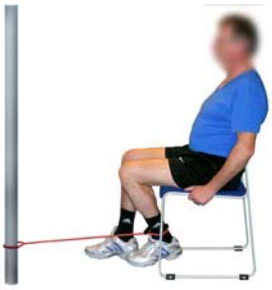

Figure 11. Knee flexors, level 2.

Exercise circle 4: Functional exercises

This circle includes exercises resembling activities of daily life.

Level 1

Exercises

A. **Chair stands:** Start in a seated position, feet parallel, putting load on both legs, slight hand support for balance
B. **Stair climbing:** Step-up (concentric muscle activation) and step-down (eccentric muscle activation) on low step-board, with or without slight hand support for balance.

Level 2

Exercises

A. **Chair stands:** Start in a seated position, feet parallel, putting load on both legs, without hand support (Figure 12).
B. **Stair climbing:** Step-up and step-down on medium high step-board, with or without bar bells.

Level 3

Exercises

A. **Chair stands:** Start in a seated position, one foot in front of the other, with or without slight hand support for balance (Figure 13).
B. **Stair climbing:** Step-up and step-down on high step-board, with or without bar bells (Figure 14).

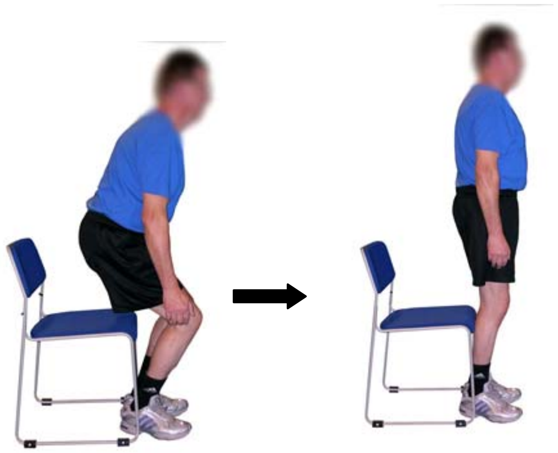

Figure 12. Chair stands, level 2.

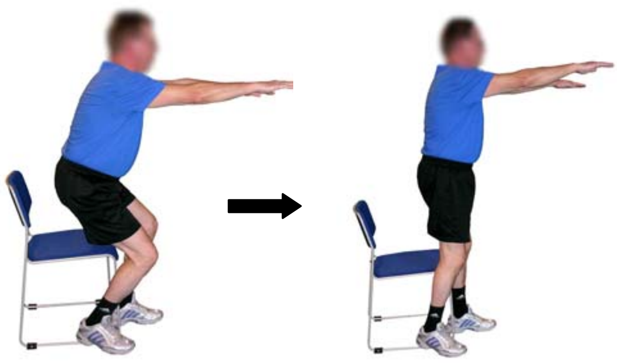

Figure 13. Chair stands, level 3.

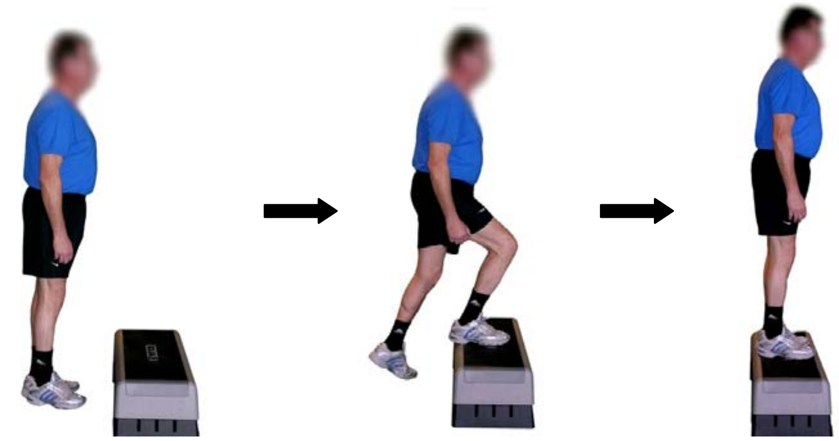

Figure 14. Stair climbing, level 3.

Part 3. Cooling down

The cooling down part consists of walking exercises forward and backwards, about 10 meters in each direction, in front of mirror (Figure 15), mobility exercises for the lower extremities (Figure 16) and stretching exercises for the lower extremity muscles for a total of about 10 minutes.

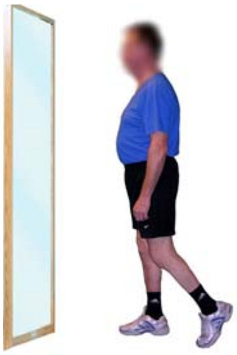

Figure 15. Walking forwards and backwards, about 10 meters in each direction, in front of mirror

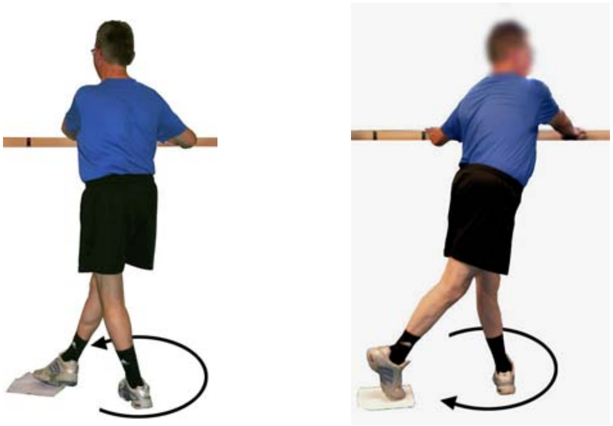

Figure 16. Mobility exercise, hip rotation during loading. Note: This mobility exercise is used for patients with hip OA, but not for patients with knee OA (because of pain and a feeling of functional instability when performing the exercise).
